# CloudMan as a platform for tool, data, and analysis distribution

**DOI:** 10.1186/1471-2105-13-315

**Published:** 2012-11-27

**Authors:** Enis Afgan, Brad Chapman, James Taylor

**Affiliations:** 1 Center for Informatics and Computing (CIR), Ruđer Bošković Institute (RBI), Zagreb, Croatia; 2 Bioinformatics Core, Harvard School of Public Health, Boston, MA, USA; 3 Department of Biology, Emory University, Atlanta, GA, USA; 4 Department of Mathematics & Computer Science, Emory University, Atlanta, GA, USA

**Keywords:** Cloud computing, Service customization, Reproducibility, Accessibility, Galaxy

## Abstract

**Background:**

Cloud computing provides an infrastructure that facilitates large scale computational analysis in a scalable, democratized fashion, However, in this context it is difficult to ensure sharing of an analysis environment and associated data in a scalable and precisely reproducible way.

**Results:**

CloudMan (usecloudman.org) enables individual researchers to easily deploy, customize, and share their entire cloud analysis environment, including data, tools, and configurations.

**Conclusions:**

With the enabled customization and sharing of instances, CloudMan can be used as a platform for collaboration. The presented solution improves accessibility of cloud resources, tools, and data to the level of an individual researcher and contributes toward reproducibility and transparency of research solutions.

## Background

Cloud computing has revolutionized availability and access to computing and storage resources. It has made it possible to provision a large computational infrastructure with several mouse clicks inside a web browser. Such availability has allowed anyone, particularly individual researchers and small labs, to gain access to the necessary compute infrastructure and apply it toward a desired domain. Coupled with the ability to provision the resources for the duration of a task, this opens new avenues of feasible research for the large data sciences.

Previously, we have developed CloudMan [1], a cloud manager that orchestrates all of the steps required to provision and control a complete data analysis environment on a cloud infrastructure, all through a web browser. CloudMan is primarily used in the context of Galaxy Cloud [2] and CloudBioLinux [3] and, along with the infrastructure, ensures a complete Next Generation Sequencing (NGS) analysis toolset is instantly available. A broad range of NGS tools is preinstalled on the provided deployment, configured with a large number of reference genomes, and integrated with the Galaxy application [4,5]. This makes it possible for any researcher to, for a modest cost, gain access to a personal infrastructure in a matter of minutes without any manual setup or configuration. CloudMan provides a high level, researcher-oriented, interface to this infrastructure.

Irrespective of how extensive the pool of tools made available through CloudMan and Galaxy is, users will inevitably develop their own tools or have needs for additional tools and data. Additionally, with the continued increase in data production (e.g., [6]), a centralized model for data analysis environments, like the public Galaxy site, is fundamentally not scalable. To overcome these issues, there is a need to shift to a decentralized model for service access. Such a model should allow individual researchers to easily utilize and reuse available tools and resources without manual configuration. Thus far, there has been a tendency to provide a custom solution for individual tools that adapts a given tool to its decentralized execution. For example, tools such as Crossbow [7], MyRNA [8], and CloudBurst [9] each provide a tailored solution for a specific problem. Such solutions not only require considerable effort to be adapted for different tools but, more importantly, make complex analyses that are composed of multiple steps by various tools difficult.

Alternatively, there are general-purpose infrastructure configuration management tools such as LCFG, CFEngine, Bcfg2, Puppet or Chef. These enable a systematic and mostly reproducible approach to configuring a set of machine images. However, besides needing to write the low-level deployment details, these tools do not provide infrastructure management options but focus on machine configuration. Tools such as StarCluster or cloudinit.d from Nimbus enable a set of underlying cloud resources to be managed as a unit. However, these tools require individual images to be manually and explicitly configured for use in life sciences, require use of command line tools, and work on only a single type of a cloud. Table 1 provides a more detailed overview of each of these projects and how those relate or differ from the topics described in this paper.

**Table 1:** A list and an overview of the pertinent projects related to the cloud infrastructure management

***Cluster provisioning and management projects and tools***	
(Sun/Oracle/Open) Grid Engine http://en.wikipedia.org/wiki/Oracle_Grid_Engine	Enables a set of computers to be composed into a compute cluster, enabling user to submit compute jobs via a unified interface. CloudMan is currently using the most recent open source version of Sun Grid Engine.
Condor http://research.cs.wisc.edu/condor/	Enables high throughput computing on distributed compute resources, providing features such as job dispatching and monitoring. We are actively looking to integrate Condor into CloudMan.
Rocks http://www.rocksclusters.org/	Focuses on compute cluster configuration and management with no application-level integration (e.g., data sources, dependent tools, scaling) or deployment bundling and sharing.
OSCAR http://svn.oscar.openclustergroup.org/	Focuses on allowing users to install, administer, and program a dedicated compute cluster with a range of installed packages, with the default set of packages focusing on scientific computing.
*Cloud orchestration projects and tools*	
Eucalyptus http://www.eucalyptus.com/	These are in many ways similar cloud middleware projects enabling Infrastructure-as-a-Service (IaaS) management of a compute infrastructure or a datacenter. These projects provide building blocks (e.g., virtual machines, block storage, object storage, networking) for assembling higher-level application services, as the one described in this paper.
OpenNebula http://opennebula.org/	
OpenStack http://www.openstack.org/	
StarCluster http://star.mit.edu/cluster/	Enables a general-purpose compute clusters to be easily deployed in the AWS environment using the command line. Although feature-full, at the moment, StarCluster operates only in the AWS context and provides no notion of deployment sharing, integration with specific applications and data sources, or a graphical management interface.
DeltaCloud API http://deltacloud.apache.org/	These provide a uniform API allowing a standardized way of programmatically communicating with a range of clouds without needing to differentiate between those. We have and are exploring options of using such libraries internally.
Libcloud API http://libcloud.apache.org/	
CloudInitD http://www.nimbusproject.org/doc/cloudinitd/	Enables a contextualization hook to be made available in a given cloud instance allowing one to customize that particular instance at runtime by providing explicit system-level instructions that should be executed by the system at boot time; such functionality is an integral part of any IaaS cloud middleware.
*Server configuration management projects and tools*	
Puppet http://puppetlabs.com/	These projects fall under the category of resource configuration management allowing one to provide a detailed recipe that is retrieved by a given machine from a predetermined server at boot time. The recipe is then executed allowing the given machine to be configured as specified in the recipe. The specified tools ensure the recipe is properly executed and a machine configured as instructed. However, once configured, these solutions do not focus on subsequent user-level application management of the infrastructure in a cohesive manner (i.e., as a compute cluster, a cooperative deployment, balancing the workload, data persistence) and thus represent a lower level of interaction with the infrastructure than what is described in our manuscript.
Chef http://wiki.opscode.com/display/chef/	
LCFG http://www.lcfg.org/	
CFEngine http://cfengine.com/	
Bcfg2 http://trac.mcs.anl.gov/projects/bcfg2	

The goal of this work is to enable reuse of the general-purpose infrastructure management and integrated tools provided by CloudMan. **We have made it possible to customize each instance of CloudMan and, if desired, preserve those customizations**. Such functionality makes it possible for a researcher to add their own tool to an instance and utilize it in conjunction with other existing tools. Similarly, a researcher can add or customize the data (e.g., reference genomes or analysis data) initially available on their instance. Once the customizations are done, it is possible to persist those changes and thus make them automatically available the next time a given cluster is started. As a result, custom and open-ended analyses can be composed with ease while utilizing the infrastructure management capabilities provided by CloudMan. Previously, it was possible to customize an instance of CloudMan but it was not possible to persist those customizations.

Instance customization allows an existing and field tested solution like CloudMan to be utilized for an individual researcher’s novel analysis. However, customized instances must either be kept alive and accessible for others wishing to use those same tools or the effort of instance customization needs to be replicated by each researcher that would like to use the same tool or data. Either of these approaches may be prohibitively expensive in financial or human effort terms, limiting the potential impact of any given customization.

To foster a fully collaborative environment, **CloudMan provides support for instance sharing**. Each CloudMan instance can be shared as a point in time configuration (in terms of tools, data, and configurations) with individuals or made public. Once shared, a user can start their own instance of the shared CloudMan instance and all of the customizations performed on the shared instance will be automatically available on the derived instance. In addition to sharing instances whose tools have been customized, researchers can share instances that have had data uploaded and analyzed. This makes it possible to share partial or complete analysis environments, allowing multiple analysis directions to be considered in parallel as well as making published analysis methods and results fully accessible. In conjunction with the Galaxy application, this ensures complete reproducibility of an analysis. It is now possible to encapsulate the compute and data storage infrastructure, the tools, and configurations into a single, independent, and shareable unit.

With the described features, **CloudMan can be utilized as a platform** for tool, data, and analysis distribution. This alleviates researchers from having to install or configure their analysis environment before utilizing it; instead, tool owners can install and configure their tools, provide sample data, and share the customizations, allowing domain researchers to test and utilize their tools with zero system-level prerequisites. By combining the strengths of CloudMan, Galaxy and CloudBioLinux, this enables a new model of decentralized research focused on scalability and reproducibility.

## Implementation

**Figure 1:**
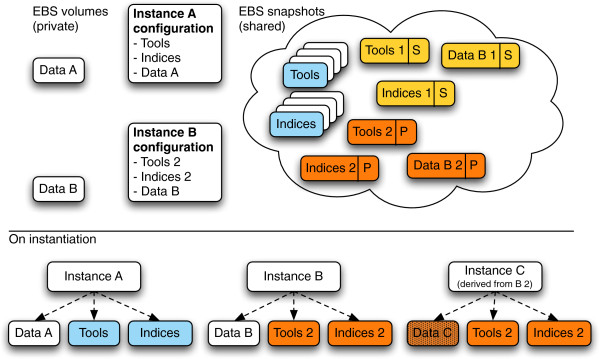
**A conceptual representation of CloudMan’s architectural components that facilitate customization and sharing of instances.** Each instance is self-contained by keeping track of the configuration components that make up the deployment. As a result, it is possible to create custom versions of the default set of tools or indices. For example, instance **A** is using the default configuration (EBS snapshots colored in blue) while instance **B** has been customized (colored in yellow and orange). Once customizations are created, they can be shared with specific users (denoted with ‘S’) or made public (denoted with ‘P’). Instances are shared as point in time data and configuration. In the shown example, instance **B** has been shared at two time points. Any derived instances (instance **C**) will use the shared instance configuration settings upon startup. Each instance has its own user data; derived instances use the shared instance’s data and build on top of it.

The CloudMan platform is currently available for instantiation on the Amazon Web Services (AWS) cloud infrastructure as part of the Cloud Galaxy application [4,5] and with CloudBioLinux. Support for private and academic clouds based on OpenStack and OpenNebula middleware exists and requires custom deployment (e.g., via CloudBioLinux configuration scripts). An example of such a deployment is available on Australia’s national cloud, NeCTAR, which is based on the OpenStack middleware. CloudMan itself is written in Python as a standalone web application and is open source licensed under the MIT license. The instructions on how to use all of the features of CloudMan and Galaxy Cloud are available at http://usecloudman.org while the source code is available at http://bitbucket.org/galaxy/cloudman. Overall, features of CloudMan that make instance customization and sharing possible are rooted in the fact that each CloudMan instance is self-contained and a complete deployment is realized at runtime; Figure 1 depicts these concepts.

### Instance customization

Each CloudMan deployment is characterized by a set of interconnected components that work in unison. At the infrastructure level, each instance is composed of *a machine image, a configuration repository,* and *one or more persistent storage units*[10].

The *machine image* represents a common denominator across all instances and contains all of the core software and libraries. Next, each instance has its own configuration repository that includes the source code for CloudMan itself, a boot-time contextualization script, references to the persistent storage resources (see below), and any application-specific configuration files. Through such a model, each instance is self-contained and customizable since it can be reestablished without external dependences. In the context of AWS, Simple Storage Service (S3) is used as the *configuration repository* and comparable object storage services for other cloud middleware.

Lastly, each instance is associated with *persistent storage resources* that store both data and applications available to the instance. Persistent storage resources are realized as a combination of data volumes and snapshots. In the context of AWS, these are the Elastic Block Store (EBS) volumes. In the context of OpenStack, these are Nova volumes. Tools that are used but not modified at runtime are stored on the snapshots. At instance runtime, those snapshots are used to create temporary volumes, which are attached to instances and used in read-only mode. These snapshots can be modified to include any desired tool. Such modifications are performed at the file system level and the process of adding a tool is the same as installing a tool on any other comparable system. Once modified, a new EBS volume is created and the instance configuration in the persistent configuration repository simply needs to point to the new snapshot and the instance will use it as part of its deployment. The process of persisting the custom cluster configuration has been integrated into CloudMan’s web interface, thus simplifying and automating this process.

Similar to the tools, the reference data associated with an instance can be modified. An instance can be modified to include custom reference datasets or sample data to run a tool. Once stored on a volume, via the CloudMan interface, a snapshot of the volume is created and the instance configuration modified to reflect the customization, making it available for future invocations.

### Instance sharing

Instance sharing leverages the fact that each instance is self-contained and makes all of its configurations and data available for derived instances. CloudMan automates this process by creating a copy of the instance’s configuration and adjusting permissions on shared objects like volume snapshots. Sharing is realized as a point-in-time snapshot of the configuration and data allowing an instance to be shared multiple times at different time points. Furthermore, each shared instance may have different access permissions set. A subtle but useful observation is that an instance can be shared with oneself, allowing exploratory customization or multiple data analysis paths to be undertaken while allowing simple reversal to a known instance state.

Once an instance is shared, it is assigned a time-point *share string*. The string is based on the cluster’s bucket name and a time stamp when the share was created. When a derived instance is desired, researchers provide the share string during the initial cluster configuration of a new CloudMan instance. CloudMan will then create a new repository based on the shared configuration, starting all of the services that were configured on the shared instance. All of the data and customizations that were performed on the shared instance will be immediately available on the derived instance. However, the two instances are separate and no data is shared between those after instantiation.

The described instance sharing is currently functional on the AWS cloud. Instance sharing on other cloud middleware solutions that the CloudMan platform is compatible with (OpenStack, Open Nebula, and soon Eucalyptus), is technically not yet possible due to the currently available cloud middleware functionality. The conceptual architecture enabling instance sharing is sufficiently versatile and compatible with the general cloud concepts to operate in these different cloud models so it is primarily a matter of time before the functionality is added. Lastly, due to the many technical details (e.g., data transfer rates), and issues with compatibility between multiple cloud providers (e.g., infrastructure building concepts), instance sharing between isolated clouds is not currently supported but is planned.

## Results

To demonstrate the reproducible and collaborative nature of CloudMan, we prepared a shared instance containing a full exome sequencing pipeline for NGS data that is available on the AWS cloud. This instance demonstrates a fully automated pipeline which processes FASTQ formatted files directly off a sequencer and produces quality filtered variant calls, alignment files and summary metrics for sequencing, hybrid selection and variant calling.

The curated and shared CloudMan instance consists of two components: preinstalled software on the utilized machine image, and the analysis data on an Amazon EBS snapshot. The machine image consists of the preinstalled tools required to complete the pipeline and an automated analysis pipeline, written in the Python programming language. The CloudBioLinux distribution contains the necessary bioinformatics software: BWA for alignment, Picard [11] for BAM format file manipulation and analysis, FastQC [12] for sequencing quality assessment, GATK [13] for alignment post-processing plus variant calling, and snpEff [14] for variant effect assessment.

The data volume attached to the CloudMan instance contains two FASTQ files that demonstrate the entire analysis process. The files are from an exome-targeted hybrid selection experiment, kindly provided by V. Mootha and S. Calvo (Broad Institute and the Center for Human Genetic Research at MGH). The processing pipeline performs alignment and variant calling, uploading resulting BAM alignments, VCF variant calls, and PDF summary files into a Galaxy Cloud instance for additional analysis.

The analysis pipeline [15] coordinates the running of this software across a parallel SGE environment setup by CloudMan. Coupled with the ability to control the number of running instances using the CloudMan console, this allows researchers to readily scale up the analysis to handle large numbers of samples.

Configuration files allow customization of the entire pipeline and analysis process. So in addition to providing a framework for learning CloudMan's sharing capabilities, the image allows researchers to directly run their exome sequencing analysis starting with just FASTQ files. This ability to both share our analysis and also directly enable re-running or custom large-scale processing is a uniquely powerful feature of the CloudMan framework. The CloudMan wiki [16] contains the instance ID for the latest version of this pipeline. This is a community-curated site that will serve as a central repository for publicly available CloudMan instances.

## Discussion

The proliferation of NGS technologies greatly increases individual researcher’s ability to access vast volume of sequence data. Cloud computing, CloudMan, and Galaxy have made considerable strides in enabling researchers to perform large-scale custom analyses. Cloud computing provides on-demand access to compute and storage resources. CloudMan bridges the low-level infrastructure components offered by cloud providers and the high-level services desired by researchers with wizard-guided compute cluster setup, automation of machine configuration, dynamic persistent storage, elastic resource scaling, and customizable, sharable instances. An instantly accessible cloud version of Galaxy eliminates many of the setup and resource obstacles to establishing a web-based analysis platform.

In the rest of this section, we describe two scenarios that highlight the benefits of CloudMan's customization and sharing functionality.

### A platform for experimentation

With the ability to customize one’s CloudMan instance, CloudMan has developed into a platform that allows reuse and promotes faster development of exploratory science. It reduces the entry level for cloud computing by combining community-developed tools with the ability to share customized analyses. Installing and configuring a tool on the CloudMan platform corresponds to that of installing a tool on any other (UNIX) machine. However, once installed, the extent in which the given tool can be utilized differs substantially: it is trivial to gain access to multiple compute nodes and execute the tool across those. Furthermore, the researcher does not need to be limited by the size and availability of the local system but can instead exploit parallel tools on multiple compute nodes. Unlike a locally available infrastructure, a cloud infrastructure boasts the option to utilize architecturally different types of worker nodes (e.g., high memory vs. fast CPU nodes) – simply start an instance of the cluster and choose a different machine type – CloudMan ensures all of the data and configurations are preserved. It is thus possible to empirically test the behavior and requirements of a tool while avoiding any complicated or repeated tool and environment setup. Overall, the ability to customize CloudMan instances eliminates the requirement for tool developers and users to own and maintain compute infrastructure while allowing full utilization of all the features cloud computing has made possible.

### A platform for collaboration

CloudMan’s ability to share individual instances shifts the availability of a fully functional and accessible analysis solution from having to be provided by tool developers and system administrators to being within reach of individual researchers. For example, it allows for the following three sample scenarios to take place:

• A user installs and configures a tool, making it available in Galaxy Once this instance is shared, others may instantiate the exact configuration of the cluster, allowing them to utilize the added tool in a matter of minutes with no configuration or installation steps required. Moreover, depending on the researcher’s needs, the size and type of the underlying infrastructure can simply be adjusted to match their current needs.

• A technician uploads some data, optionally performs initial analysis steps, and shares the instance. Bioinformaticians and bench scientists may now build on the existing data and analysis steps without having to upload the data again or perform the same initial steps, thus enabling and speeding up the data analysis process. In addition, multiple copies of the same instance may simultaneously be created, allowing different analysis steps to be undertaken in parallel.

• A researcher performs a complete analysis and, adds software and reference genome data. Once this instance is shared, its share string can be published as part of the analysis report, allowing others to instantiate the exact and complete version of the instance, including all of the customizations and the analysis data. As a result, without having to provision the resources or transfer the configuration environment to an accessible location, the analysis can be reproduced in its entirety.

**Figure 2:**
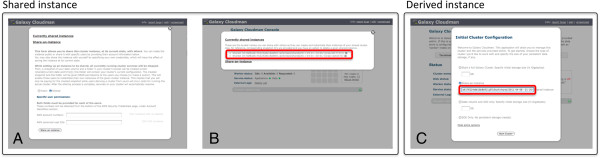
**Web interface used to share an instance and later create a derived instance.** (**A**) A user can chose to make an instance public or share it with specific user or group of users. (**B**) Once shared, the time point share is assigned a share ID string. This string is shared with users, who simply (**C**) provide the share ID string at the time of new cluster instantiation to create a derived cluster.

Figure 2 shows the web interface that is used to create and instantiate shared instances. With the described functionality, CloudMan has grown into a platform for collaboration. This functionality has broader implications that reach much further than the above sample scenarios illustrate; for example, journals could require a CloudMan shared instance that includes the tools, configurations, and utilized data to be submitted as part of the paper itself. This would allow reviewers and other researchers to not only verify the bioinformatics results but also to instantly build on the same foundation. As a result, the CloudMan platform promotes accessible, transparent, and reproducible research while greatly simplifying many of the low-level details required to manage and maintain a compute infrastructure required to perform data analysis.

## Conclusions

As technologies such as cloud computing mature, it is essential to provide easy-to-use solutions that make them accessible to researchers. With the growth in accessibility, there is also a need to minimize the amount of repeated effort required to establish custom analysis platforms. CloudMan ensures all of the low-level infrastructure management details are automated and abstracted from the user while making the underlying framework accessible for reuse. With CloudMan, a broad range of tools and reference datasets required for NGS analysis are made instantly accessible and available through Galaxy. Additional tools or data can then easily be added to individual instances while reusing all of the existing features. Once customized, a given instance can easily be shared with individual users or made public. Derived instances contain all the data and customizations that were performed on the shared instance while being independent of each other. This allows researchers to make tools, data, and analyses available and instantly accessible.

## Availability and requirements

• Project name: CloudMan

• Project home page:http://usecloudman.org

• Operating system(s): *NIX

• Programming language: Python

• Other requirements: cloud infrastructure account (e.g., AWS, NeCTAR)

• License: MIT

• Any restrictions to use by non-academics: None

## Abbreviations

AWS: Amazon Web Services; EBS: Elastic Block Storage; LCFG: Large Scale UNIX Configuration System; NGS: Next Generation Sequencing; S3: Simple Storage Service; SGE: Sun Grid Engine.

## Competing interests

Authors have no competing interests in this project.

## Authors’ contributions

EA, BC and JT conceived the project, structured the conceptual plans, implemented the software, validated the functionality, and wrote the manuscript. All authors have read and approved the final version of the manuscript.

## References

[B1] Afgan E Baker D Coraor N Chapman B Nekrutenko A Taylor J Galaxy CloudMan: delivering cloud compute clusters BMC Bioinformatics 2010 11 12 S4 10.1186/1471-2105-11-S12-S4 21210983 PMC3040530

[B2] Afgan E Baker D Coraor N Goto H Makova K Nekrutenko A Taylor J Harnessing cloud-computing for biomedical research with Galaxy Cloud Nat Biotechnol 2011 29 11 972 974 10.1038/nbt.2028 22068528 PMC3868438

[B3] Cloud Biolinuxhttp://www.cloudbiolinux.com/

[B4] Goecks J Nekrutenko A Taylor J Galaxy: a comprehensive approach for supporting accessible, reproducible, and transparent computational research in the life sciences Genome Biol 2010 11 8 R86 10.1186/gb-2010-11-8-r86 20738864 PMC2945788

[B5] Afgan E Goecks J Baker D Coraor N Nekrutenko A Taylor J Yang K Galaxy - a gateway to tools in e-science Guide to e-science: next generation scientific research and discovery 2011 London: Springer 145 177

[B6] Big data rains down on seattlehttp://www.hpcwire.com/hpcwire/2011-10-20/big_data_rains_down_on_seattle.html

[B7] Langmead B Schatz MC Lin J Pop M Salzberg SL Searching for SNPs with cloud computing Genome Biol 2009 10 11 R134 10.1186/gb-2009-10-11-r134 19930550 PMC3091327

[B8] Schatz MC Langmead B Salzberg SL Cloud computing and the DNA data race Nat Biotechnol 2010 28 7 691 693 10.1038/nbt0710-691 20622843 PMC2904649

[B9] Schatz MC CloudBurst: highly sensitive read mapping with MapReduce Bioinformatics 2009 25 11 1363 1369 10.1093/bioinformatics/btp236 19357099 PMC2682523

[B10] Afgan E Baker D Nekrutenko A Taylor J A reference model for deploying applications in virtualized environments Concurrency and Computation: Practice and Experience 2012 24 12 1349 1361 in press 10.1002/cpe.1836 PMC8073171 33907528

[B11] Picardhttp://picard.sourceforge.net/

[B12] FastQChttp://www.bioinformatics.babraham.ac.uk/projects/fastqc/

[B13] DePristo M Banks E Poplin R Garimella K Maguire J Hartl C Philippakis A del Angel G Rivas MA Hanna M A framework for variation discovery and genotyping using next-generation DNA sequencing data Nat Genet 2011 43 5) 491 498 21478889 10.1038/ng.806 PMC3083463

[B14] SnpEff: variant effect predictionhttp://snpeff.sourceforge.net/

[B15] bcbio_nextgenhttps://github.com/chapmanb/bcbb/tree/master/nextgen

[B16] CloudMan wiki https://bitbucket.org/galaxy/cloudman/wiki/SharedInstances

